# Decomposition of juvenile-sized remains: a macro- and microscopic perspective

**DOI:** 10.1080/20961790.2018.1489362

**Published:** 2018-09-20

**Authors:** Ann H. Ross, Amanda R. Hale

**Affiliations:** Department of Biological Sciences, North Carolina State University, Raleigh, NCUSA,

**Keywords:** Forensic sciences, postmortem interval, decomposition, juvenile remains, microscopic alteration, histology

## Abstract

There is currently a dearth of research investigating the progression and rate of decomposition for juvenile remains. It is thought that juveniles and infants decompose at an increased rate relative to adults due simply to body mass and that skeletal preservation is commonly dependent on intrinsic levels of bone mineral density (BMD). This study investigates the environmental variables important in driving juvenile decomposition as well as examining if currently accepted methodology for quantifying adult decomposition can be applied to juvenile remains. Furthermore, histological analysis is undertaken to test the Histological Index (HI) as a semi-quantitative indicator of decomposition. Thirty-five *Sus scrofa* ranging between 1.8 and 22.7 kg were deposited to simulate body mass of human infant and juvenile remains. Pigs were deposited every season over two years in the southeastern US with five depositional types: bagged, blanket wrapped, and surface control foetal remains, surface, and buried juvenile remains. Remains were scored quantitatively throughout soft tissue decomposition. Following study completion and skeletonization, a femur was selected from each set of remains for histological analysis. Thick sections were assessed under standard brightfield light and scored using Oxford Histological Index (OHI). Results indicate that seasonal variation is an important factor to consider even when using a standardized time variable such as accumulated degree days (ADD), particularly variation in soil moisture. Soil moisture was a consistent significant variable in the mixed effects model. The pattern of decomposition using total body score (TBS) was similar to that observed by others prior to log transformation with a rapid incline early in decomposition with levelling off. The correlation between time in days, ADD, and TBS was not as strong as those previously reported (*R*^2^ = 0.317 and 0.499, respectively) suggesting that TBS as it is currently formulated cannot be directly applied to juvenile remains. Finally, the OHI model performed moderately well, but was variable even within seasons across multiple years.

## Introduction

In forensic casework, understanding all stages of decomposition is integral to establishing time-since-death or the postmortem interval (PMI), which with victim identification can inform through exclusion or inclusion of an individual within a missing person’s pool, and increase case resolution [1]. Research has shown that decomposition is a highly variable process owing to intrinsic factors such as body mass (e.g. height and weight) of the individual [2] and extrinsic factors such as how the body was deposited and local environmental factors (e.g. temperature, soil acidity, insect activity, etc.) [3–11]. Estimating PMI is relatively accurate using early soft tissue decompositional changes that typically involve the forensic pathologist evaluating the stages of rigor mortis, livor mortis, and algor mortis to name a few. This is not the case, however, with the later stages of soft tissue decomposition and postmortem changes to the skeleton due to later taphonomic agents [12, 13]. Therefore, the estimation of PMI is cautioned against as it is the most difficult and generally seen as the most inaccurate part of the forensic anthropological examination.

PMI estimation has been largely qualitative in nature [14], which precludes quantitative assessments in actual cases; hence, there is an increasing demand for a quantitative methodology that can yield estimates with a reasonable degree of certainty [15]. Even with a transition to more quantitative research within forensic anthropology, difficulty exists in creating a universal methodology or approach because of the range of variability across environments and depositional contexts [16]. Although there is a need for an accepted standard methodology that can be adopted, the reliability of the method and its accepted validity within the discipline is still questioned. Since the National Academy of Sciences report and the criticisms of even pairing the term science with forensics, there has been an attempt to quantify most forensic anthropological methods [12, 15, 17, 18], but PMI estimation is still lagging behind. For PMI, the most employed quantitative model is based on a retrospective study of adult remains by Megyesi et al. [3] using soft tissue decomposition to score different body regions to arrive at a total body score (TBS) over accumulated degree days (ADD) or the summation of temperature over time [3].

Considering the difficulty with estimating PMI in adult remains and the dearth of data for decomposition and weathering patterns in juvenile remains, establishing the PMI for juvenile remains is problematic. The leading issue lies in the lack of comparative decomposition studies in varied depositional contexts (e.g. plastic bags and blankets). Differences in decompositional changes between adults and children have been related to overall size or greater surface-to-volume ratio [19, 20] and bone mineral density (BMD) [21]. While decreased BMD is a product of skeletal weathering due to the loss of organic material in the postmortem environment, intentional starvation, and neglect will also result in lower BMD in an infant or child prior to death [22–24]. This is complicated by an overall lower starting BMD in subadults relative to adults. The smaller size and most likely the lesser proportion of mineralized bone in subadults contributes to faster decomposition [19, 20]. Spicka et al. [25] found that different equations were needed for estimating PMI using gravesoil chemistry between neonatal and larger carcasses. The accelerated decomposition process, which can reduce a small child to a skeleton in as little as 6 d poses many challenges for law enforcement and medico-legal personnel (e.g. locating remains, establishing time-since-death, and determining cause-of-death).

### Macroscopic decomposition

The variables that have been identified as contributing factors necessitate a multi-disciplinary perspective and include but are not limited to temperature, insect activity, soil moisture, and sediment chemistry [3–5, 7, 9, 11, 26]. Temperature has been identified as a driving factor in decomposition and has been quantified as ADD to account for the covariation of time and temperature on the rate of decomposition [27]. Following temperature, soil moisture has been identified as the second most important variable in decomposition [28], whereby too much or too little moisture can delay decomposition [29].

There are also geographic differences related to the depositional site and type of concealment such as indoor versus outdoor decomposition. More localized differences also include whether remains are buried or placed on the surface [5, 30]. Kelly et al. [31] examined the effects of clothing in South Africa and found that wrapped carcasses remained in the advanced decay stage longer than unwrapped carcasses. Voss et al. [10] also found that clothed carcasses took longer to decompose, but in Western Australia, they remained in the active decay stage longer than unwrapped carcasses. Blau and Forbes [20] argue that clothing can partially negate the effects of general soil environments and delay decomposition. In addition, coverings can retain moisture, which can promote adipocere formation.

Another debated question is whether body mass affects the rate of decomposition [32]. Most studies have focused on adult decomposition as human adult cadavers are easier to acquire, as evidenced by the numerous decomposition facilities around the world based on body donation programs. However, juvenile and infant decomposition also requires investigation as they are even more likely to be intentionally concealed after death. Some studies have used smaller animals other than pigs [33] in decomposition research, and even cubes of meat [9], but no comparative synthesis has been achieved in relation to size [34]. However, Spicka et al. [25] found that carcass mass below 20 kg decomposed more rapidly than those above 20 kg and released a lower concentration of ninhydrin-reactive nitrogen over time into the grave soil than larger mass carcasses suggesting that mass does play an influential role in decomposition rates.

### Microscopic decomposition

Macroscopic changes visible during decomposition have underlying microscopic causes such as microbial action [35–41] and autolysis [42–45]. In concert, bone is perceived as an inert tissue, but has been shown to be a rather dynamic tissue as it reacts with both the burial environment and the degrading soft tissues [38, 39, 46, 47]. Thus, one promising, but under-utilized area of forensic taphonomy is microscopic evaluation of diagenetic effects on bone. Most studies assume no postmortem alteration until skeletonization [35, 46, 48], but gut bacteria, active in the early postmortem period have been shown to invade bony tissues as well [35, 37, 48, 49]. Understanding microbial alteration is important for two reasons: (1) it can aid practitioners in discriminating bones subject to contamination, and (2) it can provide useful information about taphonomic histories when the environmental and biological processes responsible for their formation are understood [35]. In forensic anthropology, where histology of bone is commonly undertaken for other analyses such as age-at-death estimation, it is important to be able to recognize any form of postmortem microscopic alteration [50].

Researchers have hypothesized that microstructural changes to bone can occur within days after death because of endogenous gut bacteria invading the skeleton with some fly associated bacteria being present early in decomposition as well [46]. This is particularly salient because gut bacteria have been shown to invade body tissues within 24 h postmortem and migrate into bone via vascular channels within a maximum of 3 d after death making it relevant to forensic contexts [46, 48, 51]. Studies designed to test microstructural alteration have had conflicting results; with some finding microstructural change within a few years after burial [52], while others find no alteration after decades of exposure [38]. However, most studies have used defleshed samples to assess diagenetic alteration, which reduces endogenous bacterial activity [53]. Bell et al. [46] were able to identify postmortem changes after as little as three months using backscattered-electron scanning electron microscopy (BSE-SEM) that could enhance areas of destruction for more precise identifications of diagenetic factors [36, 39, 54]. While this study showed the importance of the early postmortem period on bone microstructure, it was primarily limited to fluvial environments.

Thus, the research objectives in this study are multi-faceted as it investigates the factors contributing to juvenile decomposition; including environmental parameters related to seasonality, deposition, and body covering. Specifically, this study applies current methodologies of soft tissue decomposition to juvenile- and infant-sized remains in the southeastern United States. Microstructural alteration is also investigated to provide general degradation parameters visible in different depositions. The goals of this study are to identify variables that influence soft tissue decomposition, investigate seasonal influences on the rate of decomposition, and to discern patterns of microscopic alteration that may be related to bioerosion.

## Materials and methods

### Materials

Due to compositional similarities, 38 (16 juvenile and 22 foetal) *Sus scrofa* remains were used in this study as an accepted proxy of human remains and were obtained from the North Carolina State University swine farm [55]. *Sus scrofa* typically has a body mass greater than 5 kg on average, they are a readily available analog, and they provide a general eutherian mammalian model for bone anatomy and histology. Juvenile remains were simulated with juvenile pigs having a mass between 15.9 and 22.7 kg and neonatal remains were represented by foetal pigs with a mass between 1.8 and 2.7 kg. Juvenile pigs were used as a proxy for human children up to 9 years of age (15.9–22.7 kg) and foetal pigs were used as a proxy for human neonatal remains (1.8–2.7 kg). The research years of study were 2013–2015 beginning in June of 2013 with the entire study lasting 755 d. The traditional calendar for the start of each season was used as the initial day of placement. One pig per deposition was deposited each season: one juvenile was placed on the surface and one was buried, one foetal pig was placed inside a plastic garbage bag and one was wrapped in a cotton baby blanket. This allowed for a total of eight surface and eight buried juveniles, eight foetal pigs in plastic garbage bags and eight wrapped in a cotton baby blanket. Surface foetal remains were added in the winter 2013 season (totalling 6) as a comparative control. All pigs were placed immediately following euthanization and a BMD scan. All remains were enclosed in cages to mitigate scavenging. However, despite best efforts, the bagged and control foetal remains of the winter 2013 season and the fall 2013 control foetal remains were consumed by scavengers leaving a total of 35 pigs for the study.

Deposition seasons were summer, fall, winter, and spring and the average temperatures classify this region with a Cfa climate according to the Köppen–Gieger climate classification [56]. A Cfa climate is considered temperate, without a dry season, and a hot summer. This climate class comprises 13.4% of the climatic variation in North America [56]. Weather data were collected from the State Climate Office of North Carolina Lake Wheeler Road Field Lab weather station located one-quarter mile from the field site. Data are freely available for download on their website. The variables collected were daily maximum temperature, daily minimum temperature, daily precipitation, relative humidity, soil temperature, and soil moisture.

### Scoring methodology – macroscopic analysis

Only four of five depositions (surface juvenile, bagged foetal, blanket foetal, and control surface foetal remains) were considered for soft tissue decomposition. Buried juvenile remains were left undisturbed for the duration of the study. Decompositional information was recorded using the Megyesi et al. [3] TBS approach. This method has been shown to have high inter-observer reliability among practitioners [57]. Each body region (head, trunk, and limbs) was scored separately and the TBS was calculated. All pigs were scored until skeletonization was complete. While insects are a driving factor in decomposition, fly activity was only recorded as a categorical variable, which included the presence or the absence of adults, eggs, larvae as well as beetle activity as the primary purpose of this study was to assess diagenetic changes post skeletonization. Insect colonization of concealed and non-concealed juvenile remains has been addressed separately by the entomology team at North Carolina State University [58].

### Scoring methodology – microscopic analysis

Histological thick sections were sampled from a femur taken from each of the study pigs, excluding the three foetal controls (*n* = 32) used in the study. Preparation of the histological samples followed published methods [59]. The samples were embedded in plastic resin to preserve the sample and ensure sample integrity during slide preparation. One-millimeter thick sections were produced using a *Buehler Isomet 1000* (Buehler, Lake Bluff, IL) saw with a 15 high concentration (HC) diamond-edged blade. Each thick-section wafer was ground to a final thickness of 50–75 µm on a Buehler™ variable-speed grinding unit (Buehler, Lake Bluff, IL) with a diamond disc. Each thin-section was mounted on a glass slide with coverslip using SECUREMOUNT mounting media (Buehler, Lake Bluff, IL). The following information was recorded on each slide: (1) slide identifier, (2) element name, (3) element side, and (4) anatomical orientation. One thick section per bone was produced for 32 pigs (32 midshaft femoral thick sections).

**Figure 1.**
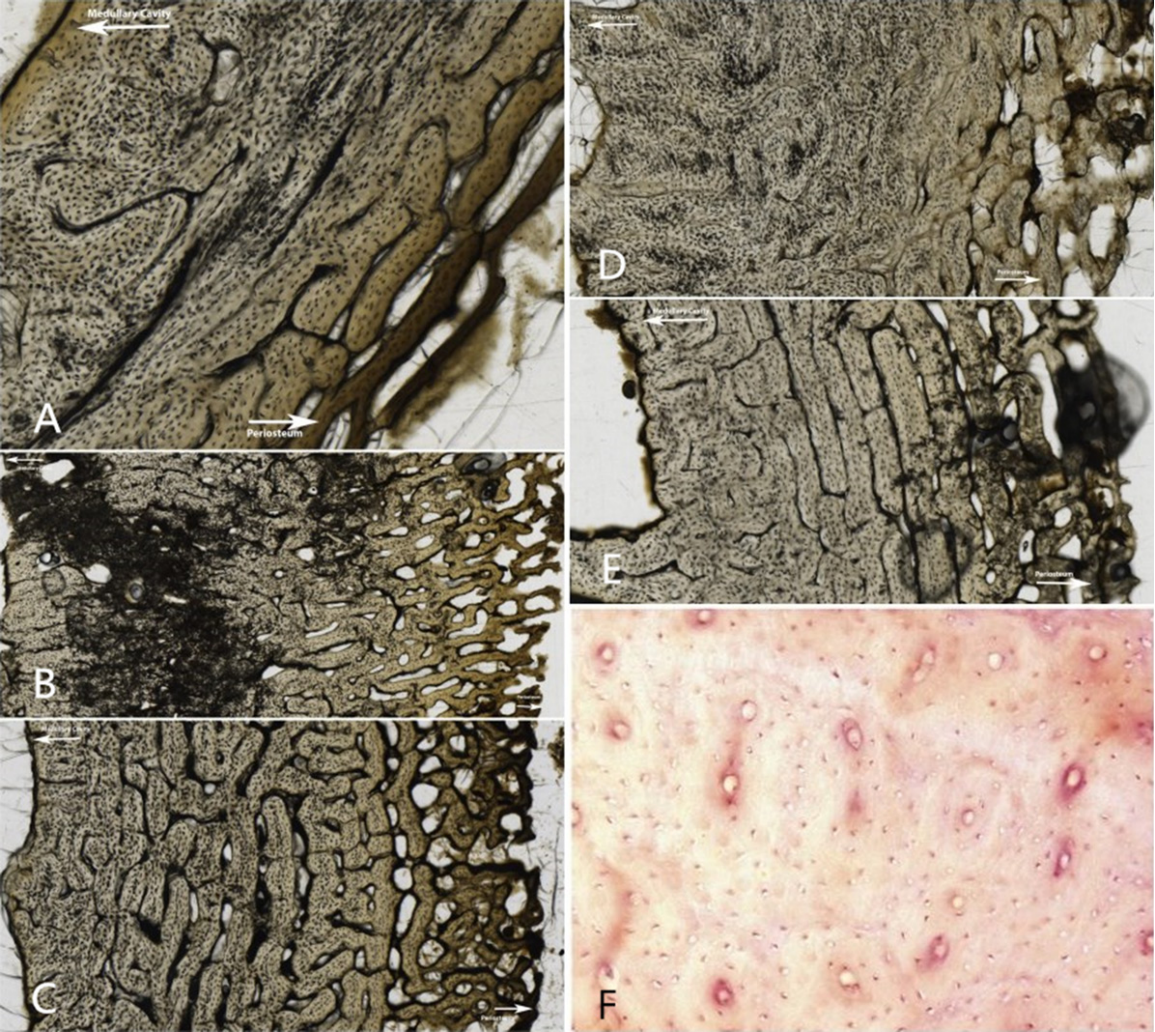
Descriptions of the Oxford Histological Index (OHI) and examples from the sectioned specimens in this study. (A) No original features identifiable, other than Haversian canals (Index 0); (B) small areas of well-preserved bone present, or some lamellar structure preserved by pattern of destructive foci (Index 1); (C) clear lamellate structure preserved between destructive foci (Index 2); (D) clear preservation of some osteocyte lacunae (Index 3); (E) only minor amounts of destructive foci, otherwise generally well preserved (Index 4); (F) Very well-preserved, virtually indistinguishable from fresh bone (Index 5) (Source: Mescher AL: *Junqueira’s Basic Histology: Text and Atlas 12th Edition*: http://www.accessmedicine.com Copyright^©^ The McGraw-Hill Companies, Inc. All rights reserved).

Histological sections were evaluated for bioerosion using a standard brightfield light as it produced better results than the recommended polarized light to assess the degree of diagenetic change and the Histological Index (HI) was employed as described by Hedges et al. [47]. Porosity and histological bone integrity were assessed with the HI, also referred to as the OHI, which assigns a value from 0 to 5 to summarize the degree of diagenetic change to bone. Figure 1 provides the description of each category defined by Hedges et al. [47] as well as an example from a representative sample in this study. This excludes an example for stage 5 as no sections were scored as well preserved in this study.

### Data analysis

ADD was the time variable employed in this study. ADD allows for comparisons across environmental regions [27, 60]. Specifically, the degree day index is calculated following Megyesi et al. [3] with the sum of the average of the minimum and maximum temperature of each day:

(1) $${ADD}_{\text{total}}=\;\sum_{t=1}^{n}\left[{(T}_{min}+\right.T_{\text{max}})/2].$$

where *T*_min_ and *T*_max_ represent the daily minimum and the maximum air temperature, *t* represents the time and *n* represents the number of days [27, 60].

A mixed random effects model, which is useful for analyzing repeated measures, was used to examine the relationship between the dependent (ADD) and independent variables (TBS, relative humidity, daily precipitation, soil temperature, soil moisture, and deposition). TBS, relative humidity, daily precipitation, soil temperature, and soil moisture were considered as fixed effects, while deposition was considered as a random effect. In addition, a simple linear regression was performed to examine the correlation between time in days (PMI) and both TBS and ADD.

A destructive degradation model was applied to examine bioerosion. This procedure is used to model product deterioration over time. A loglogistic distribution was chosen as it is more appropriate for decomposition studies that exhibit logistic patterns. This distribution examined the relationship between the response or degradation measure (HI) and time variable (ADD). The common path with intercept model was selected that fits a single distribution whose location parameter changes linearly over time [61]. All statistical analyses were conducted in JMP 13.0 [62]:

(2) $$\mu =b_{0}+\;b_{1}\;\times \;f\left(\text{time}\right).$$

where *µ* represents the mean observations, *b*_0_ represents the slope of the distribution, *b*_1_ represents, the HI and *time* represents the ADD measure.

## Results

### Macroscopic analysis

Table 1 presents the mean values at skeletonization for each deposition by season. In addition, Table 1 provides the mean values for temperature, relative humidity, daily precipitation, and soil moisture averaged over the 2-year study period. For fall and spring, the surface juvenile did not reach skeletonization until almost twice the ADD as the foetal remains. However, for the summer and winter months, the surface juvenile fell within the range of the foetal remains. The pattern observed shows the overall fastest rate of decomposition in the summer, followed by the fall season, with spring and winter seasons showing similar ADD values. Interestingly, this does not completely correspond with average temperatures as the fall season has lower average temperatures than the spring season. In addition, this pattern does not coincide with relative humidity values with humidity decreasing after the fall season.

**Table 1.** Mean values for accumulated degree days (ADD) at skeletonization [excluding one winter juvenile that remained mummified] of the blanket foetal, bag foetal, control foetal, and surface juvenile depositions as well as mean temperature, relative humidity, daily precipitation, and soil moisture by season averaged over the 2-year study period.

Season	ADD				Temperature (*n* = 56, °C)	Relative humidity (*n* = 56, %)	Daily precipitation (*n* = 56, cm)	Soil Moisture (*n* = 56, m^3^/m^3^)
	Blanket *(n* = 8)	Bag *(n* = 8)	Control *(n* = 6)	Surface *(n* = 8)				
Fall	397.7	364.2	221.8	715.8	15.0	71.931 0	0.337 8	0.287 0
Spring	867.2	1 094.6	792.6	1 565.0	23.4	61.367 3	0.323 3	0.326 1
Summer	396.5	573.6	311.7	478.5	24.6	69.968 4	0.412 5	0.157 5
Winter	1 113.3	826.9	637.2	1 167.7	11.6	65.266 7	0.562 6	0.264 9

The results from the mixed random effects model showed that for the fall season deposition and relative humidity were not significant effects (*deposition* (bag, blanket, control) degrees of freedom of the numerator (DFNum) = 3, degrees of freedom of the denominator (DFDen) = 165, *F* = 0.107, *P* = 0.956; *relative humidity* DFNum = 1, DFDen = 165, *F* = 57.469, *P* = 0.470). However, all other variables were significant at the <0.000 1 level (TBS, daily temperature, daily precipitation, soil temperature, and soil moisture). For the spring, all variables were significant (0.000 1–0.01 level) except for the control (*P* = 0.216). The summer yielded a different pattern with only three significant effects (*TBS* DFNum = 1, DFDen = 149, *F* = 34.96, *P* ≤ 0.000 1; *soil temperature* DFNum = 1, DFDen = 149, *F* = 8.17, *P* ≤ 0.004 9; *soil moisture* DFNum = 1, DFDen = 149, *F* = 25.41, *P* ≤ 0.000 1). In winter, the pattern differed with only TBS, deposition (blanket), and soil moisture having significant effects (TBS DFNum = 1, DFDen = 201, *F* = 386.4, *P* ≤ 0.012 5; *deposition* (blanket) DFNum = 3, DFDen = 201, *F* = 3.71, *P* ≤ 0.000 1; *soil moisture* DFNum = 1, DFDen = 201, *F* = 70.87, *P* ≤ 0.000 1). The simple linear regression model to compare the correlations showed a weak correlation for ADD and TBS (*R*^2^ = 0.353 6) and TBS and PMI or time since deposition (*R*^2^ = 0.211).

### Microscopic analysis

**Figure 2.**
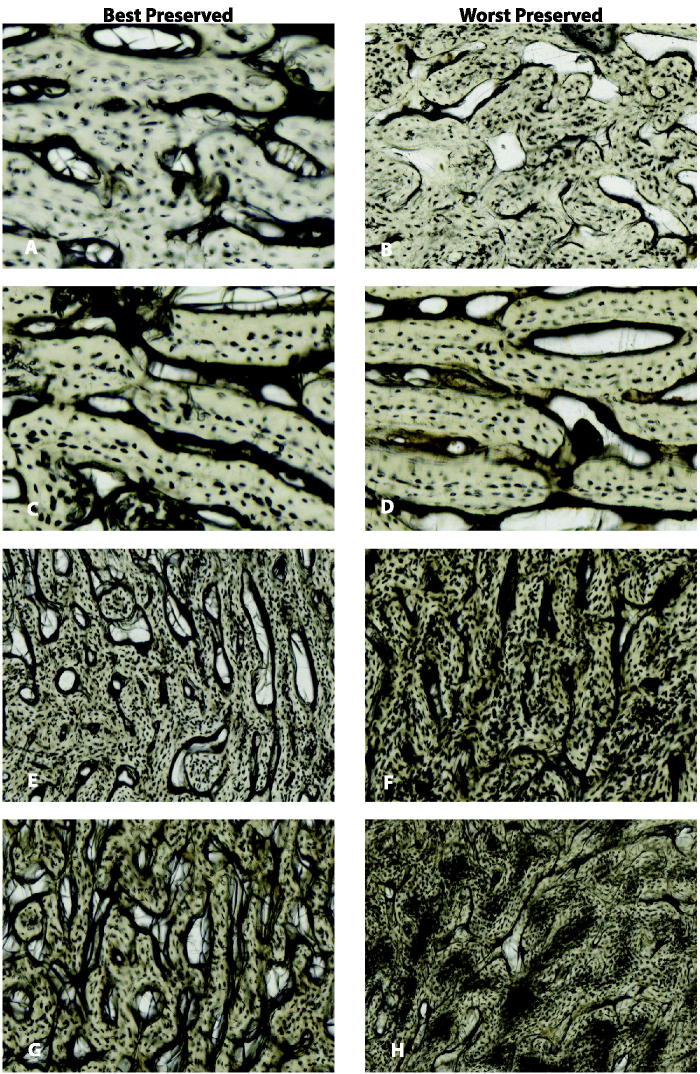
Best and worst preserved for each deposition (A) and (B) are the bagged foetal remains with an OHI of 2 and 1, respectively. (C) and (D) are the blanket foetal remains with an OHI of 3 and 1, respectively. (E) and (F) are the buried juvenilies with an OHI of 4 and 0, respectively. (G) and (H) are the surface juveniles with an OHI of 4 and 1, respectively.

Table 2 presents the probabilities calculated by the destructive degradation model and Bayesian information criterion (BIC) of each loglogistic model. The destructive degradation model results show that there is a positive linear relationship between HI and ADD for all depositions. However, observed diagenetic changes were limited to the periosteal envelope. For the bagged foetal remains, the degradation profile shows that the predicted OHI is 1.29 with set ADD 2 153.85 with a 95% prediction interval of 0.60–2.79. The crossing time distribution profile shows that there is a 67% probability that the HI score will be 1.5 at 2 153.85 ADD. For the blanket foetal remains, the degradation profile shows that the predicted OHI is 1.67 with set ADD 2 153.85 with a 95% prediction interval of 0.76–3.70. The crossing time distribution profile shows that there is a 70% probability that the HI score will be 2 at 2 153.85 ADD. For the buried juvenile remains, the degradation profile shows that the predicted OHI is 1.55 with set ADD 7 153.78 with a 95% prediction interval of 0.49–4.92. The crossing time distribution profile shows that there is a 69% probability that the HI score will be 2 at 7 153.78 ADD. For the juvenile surface remains, the degradation profile shows that the predicted OHI is 2.6 with set ADD 2 153.85 with a 95% prediction interval of 0.85–7.92. The crossing time distribution profile shows that there is a 47% probability that the HI score will be 2.5 at 2 153.85 ADD. The model performed the best with the blanket foetal remains with a 70% probability that at 2 153.9 ADD the OHI would be 2 or moderately preserved. Figure 2 illustrates the best and well-preserved sections from each deposition. None of the sections were scored as fresh, but juveniles showed the best preservation overall with one section of both buried and surface remains scoring a 4. Most scores were between 1 and 3. Table 3 provides the model from the destructive degradation profile of each deposition.

**Table 2.** Probabilities determined by destructive degradation model for each deposition and the Bayesian information criterion (BIC) of each loglogistic model. It reads that there is a 67% probability that the Oxford Histological Index (OHI) of bone would be between 1 and 2 for an accumulated degree days (ADD) of 2 153.9.

Deposition	Probability	OHI	ADD	Time equivalent^a^	BIC
Bag foetal	0.67	1.5	2 153.9	6 months	15.392
Blanket foetal	0.70	2	2 153.9	6 months	20.947
Buried juvenile	0.69	2	7 153.8	1 year	24.374
Surface juvenile	0.47	2.5	2 153.9	6 months	32.632

^a^The time equivalent for ADD in this study.

**Table 3.** Provides the model from the destructive degradation profile of each deposition with loglikelihood of each model.

Deposition	Model	Standard deviation	Log-likelihood
Bag foetal	OHI =0.457 + [−9.384×10^−5^] × ADD	0.062	4.777
Blanket foetal	OHI = -0.166 + [3.159×10^−3^] × ADD	0.065	7.354
Buried juvenile	OHI =1.081 + [−8.954×10^−5^] × ADD	0.097	9.268
Surface juvenile	OHI =0.797 + [7.288×10^−5^] × ADD	0.092	13.197

## Discussion

### Macroscopic decomposition

Broadly, the results suggest seasonal and depositional variation in decomposition related to temperature, moisture, and covering. The seasonal variation observed in this study is similar to that detected by Meyer et al. [11] with summer having the highest gross carcass decomposition. In the present study, bagged foetal remains showed delayed decomposition in comparison with the blanket and surface for all seasons except fall, which coincided with days to skeletonization. However, this is not surprising as it has been shown that plastic waste sacks slow decomposition because they limit insect access to the remains [63]. In another study by Cammack et al. [58] in the same North Carolina environment as the present study, tested decomposition of concealed and exposed juvenile sized porcine remains placed in a simulated attic environment. They found significant differences between colonizing insect species between concealed and non-concealed remains, between seasons, and depending on level of concealment, insect colonization was delayed 35–768 h [58]. For the present study, the average time of skeletonization for the bagged foetal remains was 17.5 d and 20.5 d for the foetal remains wrapped in a baby blanket.

The surface juveniles show an increased rate of decomposition for the seasons with the greatest relative humidity. This corresponds across all depositions with those fastest to decompose having the highest relative humidity values. The mixed effects model results indicate that soil moisture is a dominant driver in decomposition supporting the study by Carter et al. [29] and demonstrate that it may be the most dominant factor as soil moisture was a significant variable regardless of season. However, this is not a surprising finding as temperature and water drive most chemical reactions as well as the motility of microorganisms. In addition, fall and spring results show daily precipitation was significant in determining the rate of decomposition. In conjunction with the averages presented in Table 1, precipitation was significant in the mixed effects model for the highest precipitation in the fall season and the lowest precipitation in the spring season suggesting that moisture effects may have a threshold where too much or too little moisture have opposing effects. This corroborates the results from Carter et al. [29] that moisture has a dominant effect, but likely only within a particular range of values.

In this study, fly activity was only found to be a significant effect in the winter season suggesting inhibition of blow fly activity due to cold temperatures may have more impact than accessibility. Considering blow flies require a minimum of 10 °C for activity [64] their activity would have been impeded if not inhibited. This is in contra to the findings by Simmons et al. [65] that found when all other variables are controlled, access to remains by insects was the primary determinant of the rate of decomposition. This is mirrored in the deposition results as well, where deposition was only significant during the fall and summer seasons, which are the warmest two seasons in North Carolina. The significant effect of deposition during fall and summer indicates that delayed accessibility by insects plays a role in overall decomposition progress. In early fall season, temperatures continue to warm coinciding with decomposition prior to skeletonization in North Carolina. However, spring season showed the opposite pattern. This appears to indicate that the early seasonal period has a greater impact on the progression of decomposition than overall seasonal average temperatures, which is apparent by earliest observed ADD values at skeletonization being in fall and summer seasons.

When only the surface control foetal and juvenile remains are considered, carcass mass appears to be an influencing factor. The surface juvenile took approximately twice the temperature accumulation to reach skeletonization than the surface foetal control supporting findings by Matuszewski et al. [2] that carcass mass was significant across all treatments and similarly to the present study, while covering was a negligible variable. Coverings only impact was in the active decay stage, which mirrors the results found by Voss et al. [10]. This is specifically noteworthy as delayed discovery due to concealment of juvenile victims of homicide would impact the medicolegal investigation and case resolution [66].

The simple linear model results indicate that ADD only accounts for approximately 35% of the decomposition progressions signalling that temperature is not the primary predictive variable driving decomposition in juveniles in the southeastern US. However, Megyesi et al. [3] found that ADD accounted for 85% of the rate of decomposition, which implies that either TBS is not an appropriate scoring system for juvenile remains or that there is a difference in environment that precludes the applicability of TBS outside of the climate it was developed in. The latter is supported by Cockle and Bell [28] who found a maximum of 53% of decomposition could be accounted for by the accumulation of temperature in Canada further supporting a regional approach to PMI estimation that has been prescribed by many [6–8, 11].

### Microscopic decomposition

The results presented here indicate that microscopic destruction is seen early in the postmortem period and this would impact bone quality and subsequent inferences such as DNA testing [35, 37]. Bioerosion differed slightly by depositional mode with buried remains showing the widest range of microscopic alteration (OHI of 0–4) including the best and worst preserved specimens of any depositional type. It must be noted, however, that buried remains included juveniles or those with larger mass.

The variation observed between the buried and other deposition samples reject the notion that a universal postmortem formula can be applied. Bagged remains showed the least amount of variation, but consistently had the worst preserved specimens (OHI 1 and 2) signifying that something about the bag ecology hastens diagenetic changes likely related to microbial mobility [29]. Observations made during data collection imply that condensation inside the bag (i.e. moisture) that has been associated with increased microbial mobility could be a contributing factor for plastic bag bioerosion [29].

The blanket foetal and surface juvenile remains showed similar alterations, which could potentially make them indistinguishable in a forensic context. The surface juveniles showed a slight positive trend between ADD and HI, while the blanket foetal remains showed an almost non-linear relationship between ADD and HI. Bagged remains showed the most consistent relationship between ADD and HI with a slightly inverse relationship with greater ADD having smaller HI values. Buried juveniles also showed an almost non-linear relationship between ADD and HI. Blanket foetal remains and surface juveniles showed a positive linear relationship between HI and ADD. In this study, diagenesis was exclusively observed on the periosteal envelope suggesting an external source of microbial invasion. Future directions of this study will include histological examinations of both ribs and inominates and to compare these to the results presented here on the femora. These skeletal areas would be expected to have an increase in intrinsic microbial action within the trabecular bone resulting from putrefaction.

## Conclusion

The results of this study found that decomposition in juvenile sized remains is driven by body mass, temperature, soil moisture, and method of concealment showing seasonal variations. Fly activity was only a significant variable when inhibited during the winter season. In addition, larger pigs took approximately twice as long to decompose than those with smaller mass. Histological analysis suggests that bioerosion is a reasonable predictor of PMI using a destructive degradation model, but it is dependent upon mode of deposition. Finally, TBS does not appear to be an adequate scoring protocol for juvenile remains. The results of this study further support the importance of seasonal, geographic, and deposition specific indices for estimating the PMI.
